# Predictive models for the risk and prognosis of bone metastasis in patients with newly-diagnosed esophageal cancer: A retrospective cohort study

**DOI:** 10.3389/fsurg.2022.1014781

**Published:** 2023-01-13

**Authors:** Bei Yuan, Haojie Lu, Dong Hu, Kai Xu, Songhua Xiao

**Affiliations:** Department of Orthopaedics, Beijing Tsinghua Changgung Hospital, School of Clinical Medicine, Tsinghua University, Beijing, China

**Keywords:** bone metastasis, esophageal cancer, nomogram, overall survival, prognosis

## Abstract

**Background:**

Esophageal cancer (EC) is a common malignant tumor worldwide, and patients with both EC and bone metastasis (BM) have a poor prognosis. We aimed to determine the risk and prognostic factors for BM in patients with newly diagnosed EC and to conduct two nomograms to predict the probability of BM and overall survival after BM.

**Methods:**

Data from patients with EC from 2010 to 2015 were reviewed in the Surveillance, Epidemiology, and End Results (SEER) database. We divided participants into training and validation cohorts using univariate and multivariate logistic regression analyses and Cox regression models to explore the risk and prognostic factors of BM, respectively. Moreover, two nomograms were developed for predicting the risk and prognosis of BM in patients with EC. Then we used receiver operating characteristic curves, decision curve analysis, and calibration curves to evaluate the nomogram models. The overall survival of patients with EC and BM was analyzed using the Kaplan-Meier method.

**Results:**

A total of 10,730 patients with EC were involved, 735 of whom had BM at the time of diagnosis. Histologic type, sex, age, N stage, primary site, liver, lung, and brain metastases, and tumor differentiation grade were identified as independent BM risk factors. Histological type, chemotherapy, brain, liver, and lung metastases were identified as prognostic risk factors for patients with EC and BM. We developed diagnostic and prognostic nomograms according to the results. Receiver operating characteristic curves, calibration, and Kaplan-Meier curves, and decision curve analysis all indicated that both nomograms had great clinical predictive ability and good clinical application potential.

**Conclusions:**

Two novel nomograms were constructed to predict the risk and prognosis of BM in patients with EC. These prediction models can effectively assist clinicians in clinical decision-making based on their good accuracy and reliability.

## Introduction

Esophageal cancer (EC) ranks ninth among the most common cancers worldwide, accounting for 3.2% of 35 major cancers and approximately 5.3% of all cancer-related mortality (1). Globally, 316,000 new cases of EC are diagnosed each year with 286,000 deaths, while 13,000 new diagnoses and 12,600 deaths occur in the United States alone (2). The prognosis is poor, especially with metastatic disease (3–5). Patients with metastatic EC have a median survival time of only 6 months, and the 2-year survival rate is about 11.8% (6).

Bone is one of the most common metastatic sites of malignant tumors and the third most common metastatic organ in patients with EC (7–9), following the liver and lung (10, 11). Several studies have reported a rate of 5.2%–7.7% of bone metastasis (BM) in patients with EC, with BM accounting for 15.3%–23.6% of metastatic EC (3, 12, 13). Sex, tumor location, tumor, node, metastasis (TNM) stages, and liver, lung, and brain metastases have been correlated with occurrence of BM (14, 15). In addition to the above factors, age, pathological tumor type, blood tumor markers, and marital status are correlated with the prognosis of patients with EC and BM (3, 14).

Once BM occurs, there are few treatment options available that can effectively improve the survival of patients with EC (16, 17). Patients may present with a range of skeletal-related symptoms, including pathological fractures, hypercalcemia, pain, and nerve compression syndromes (18). For those patients with EC and BM, overall survival (OS) is poorer than that for patients with EC and other metastatic sites (4). Despite the importance of accurate prognosis prediction, the classic TNM staging system depends solely on three pathological indicators, including tumor invasion, lymph node infiltration, and distant metastasis, while ignoring other prognostic factors, leading to reduced prognostic prediction accuracy for patients with EC (19, 20). Consequently, it is essential to establish a tool that can integrate clinicopathological and other prognosis-related factors to accurately predict the prognosis of patients with EC.

Nomograms are tools that integrate multiple clinical, pathological, demographic, and oncological factors to predict the occurrence of medical events and have a great application potential for prognostic prediction of patients with cancer (21). In addition, nomograms have an advantage in accuracy over the traditional TNM staging system in intuitively estimating the survival rates of patients with cancer (22). By analyzing data from the Surveillance, Epidemiology, and End Results (SEER) database, we aimed to develop two novel nomograms to separately and quantitatively predict the risk of BM and OS of patients with EC and BM to assist clinical decision-making.

## Patients and methods

### Data source

A retrospective cohort study was used in this research. We used data extracted from the SEER database, which is comprised of data from 18 registries across the United States, accounting for 28% of the US population (23). Thus, the sample size of our study was sufficient to exclude the bias caused by factors such as region and living habits and to reach a conclusion. Because the data in this database is anonymous and public, informed consent was waived and medical ethics reviews was not required.

### Participants

**Figure 1:**
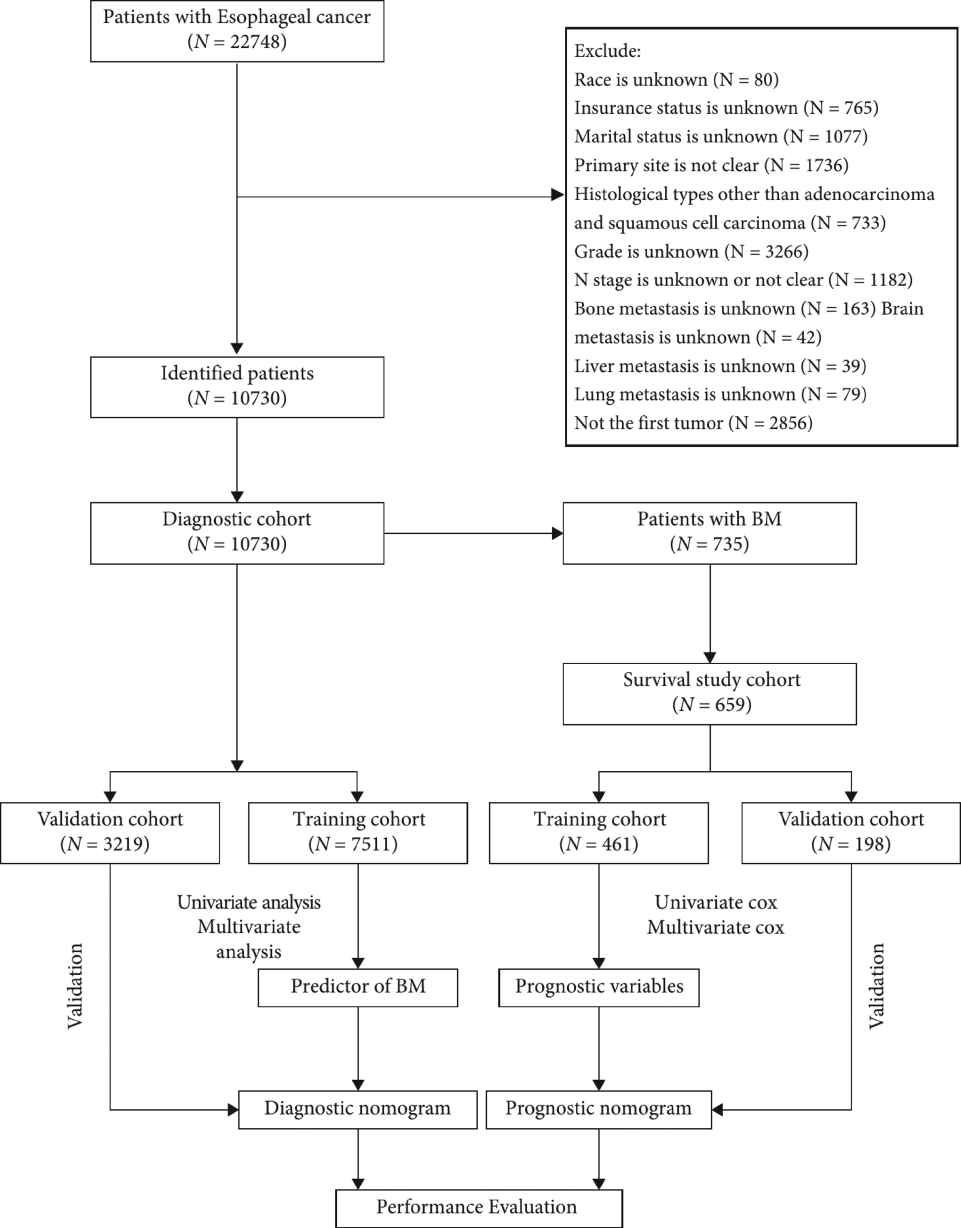
Flowchart of patient screening process and workflow of this study.

Patients who met the following criteria were included: (1) patients were diagnosed with EC between 2010 and 2015, (2) EC was the primary malignant tumor, and (3) diagnosis was confirmed by histological biopsy. Patients were screened according to the exclusion criteria below: (1) unknown insurance status, (2) unknown race, (3) unknown marital status, (4) unclear primary site, (5) histological types other than adenocarcinoma (AC) and squamous cell carcinoma (SCC), (6) unknown grade, (7) unclear or unknown node (*N*) stage, (8) unknown BM, (9) unknown brain metastasis, (10) unknown liver metastasis, (11) unknown lung metastasis, (12) EC was not the first tumor. All patients were enrolled in the diagnostic cohort to determine risk factors for BM. Patients with BM and complete treatment records, including radiotherapy, chemotherapy, and primary tumor surgery, and who survived ≥1 month were included in the survival cohort. In a 7:3 ratio, participants were randomly assigned to the training and validation cohort. Figure 1 shows the patient screening process and workflow of this study.

Internal validation was also performed. Participants in the validation and training groups were recruited from the same population, followed the same inclusion and exclusion criteria, and had consistent diagnostic and prognostic judgment criteria. We use the chi-square test to identify statistical differences between the validation and training groups for each variable, and, if identified, the groups were regrouped until the differences were not statistically significant. As this investigation was a retrospective cohort study, a blind method was not adopted.

### Predictors and outcome

To determine risk factors for BM, twelve variables were selected, including age, sex (female or male), insurance status (uninsured or insured), race (white, black, Asian or Pacific Islander, or American Indian/Alaska Native), marital status (married or unmarried), primary site (upper third esophagus, middle third esophagus, lower third esophagus, or overlapping lesion), histologic type (SCC or AC), tumor differentiation grade (grade I, well differentiated; grade II, moderately differentiated; grade III, poorly differentiated; or grade IV, undifferentiated and anaplastic), N stage (N0, N1, N2, or N3), and brain, liver, and lung metastases (yes or no). Tumor (T) stage was not used as a predictor because this data was not available for more than 20% of the patients. The diagnostic criteria for BM were based on imaging and pathological findings, and the outcomes were labeled in the SEER database.

In addition to the variables mentioned above, we also incorporated therapeutic measures to identify prognostic factors for patients with EC and BM, including chemotherapy, radiotherapy, and surgery for primary tumors. In the survival analysis, the main observation index was OS, representing the date from the diagnosis of EC to death or loss of follow-up (24). Detailed data for OS is available in SEER database.

### Development and validation of nomograms

We used univariate logistic analysis to preliminarily identify risk factors for BM, and when the *P*-value was less than 0.05, the corresponding variables would be incorporated in the multivariate logistic analysis to further verify independent BM risk factors in patients with EC. We then established a nomogram and calculated individual risk scores to determine the probability of BM. The scale at the top illustrates the points of each predictor, ranging from 0 to 100. By drawing a vertical line, each predictor can be determined with the corresponding value on the scale. The sum of the values for each predictor is displayed as the total points, which corresponds to the probability of BM shown at the bottom of the chart. To show the prediction efficiency of the nomogram, we created a receiver operating characteristic (ROC) curve and calculated the area under the curve (AUC). Decision curve analysis (DCA) was also performed and calibration curves were plotted. DCA is a common method to assess the clinical application value of a novel diagnostic tool by calculating the corresponding net benefit at each threshold probability (25, 26). The farther the curve is from the two intersecting lines, the greater the clinical application value. Calibration curves were used to evaluate differences between the actual and predicted data to determine the accuracy of the nomogram. To further assess the performance of this nomogram, ROC curves along with the corresponding AUC value, DCA, and calibration curves were also performed for the validation cohort.

Survival analyses were performed to determine the prognostic factors for patients with EC and BM. At a ratio of 3:7, participants with BM were randomly assigned to the validation and training groups. Univariate Cox proportional hazards regression analysis was preliminarily performed to investigate the OS-related factors. Then, multivariate Cox regression analyses were used on factors with *P* values less than 0.05 to identify the independent prognostic risk factors for patients with EC and BM. Subsequently, based on the results above, a nomogram was developed for predicting the OS of patients with EC and BM. The survival probabilities of patients at 3, 6, and 12 months were obtained by calculating the points in each dimension. Additionally, time-dependent ROC curves at 3, 6, and 12 months, along with the corresponding time-dependent AUC values, were plotted to demonstrate the prediction efficiency of the prognostic nomogram. Moreover, calibration and DCA curves were established. In order to compare with the training group, the validation group was also conducted with the same operation and plotted the corresponding time-dependent ROC, calibration, and DCA curves. Moreover, patients with EC and BM were classified into high- and low-risk groups according to the prognostic scores calculated by the nomogram. Kaplan-Meier (K-M) survival curves were plotted to present the survival differences between the two groups.

### Data analysis

We used SPSS (version 23.0, IBM Corp., Armonk, NY, USA) and R software (version 4.1.2, R Foundation for Statistical Computing, Vienna, Austria) to perform all statistical analyses. Comparisons of categorical data were analyzed through the chi-square test and Fisher's exact probability method, while continuous data were processed using Student's t-test or the Mann-Whitney U test according to the data distribution. *P*-values less than 0.05 were regarded as statistically significant. The confidence interval (CI) was set at 95%.

## Results

### Participants

A total of 10,730 EC patients were incorporated, and 735 patients developed BM. Table 1 presents the clinicopathological information of the participants. Demographically, 8,643 were men (80.6%) and 9,120 were white (85.0%), 6,219 patients (58.0%) were married. The median age was 66 years, and patients with BM were younger than those without BM. In terms of oncological characteristics, EC occurred more frequently in the lower third of the esophagus (7,456 patients, 69.5%), followed by the middle and upper third of the esophagus. Grades III and IV were the most common differentiation grades (5,494 patients, 51.2%), and AC occurred more frequently among all pathological types (7,475 patients, 69.7%).

**Table 1:** Clinicopathological characteristics of patients with esophageal cancer.

Variables	Level	Overall (*N* = 10,730)	Without BM (*N* = 9995)	With BM (*N* = 735)	*P* Value
Age(y)^a^	/	66(58,74)	66 (58,74)	63 (56,70)	<0.001
Sex	Female	2,087 (19.5%)	1,998 (20.0%)	89 (12.1%)	<0.001
	Male	8,643 (80.5%)	7,997 (80.0%)	646 (87.9%)	
Insurance status	Uninsured	361 (3.4%)	328 (3.3%)	33 (4.5%)	0.080
	Insured	10,369 (96.6%)	9,667 (96.7%)	702 (95.5%)	
Race	Black	1,036 (9.7%)	968 (9.7%)	68 (9.2%)	0.048
	White	9,120 (85.0%)	8,497 (85.0%)	623 (84.8%)	
	American Indian/Alaska Native	64 (0.6%)	54 (0.5%)	10 (1.4%)	
	Asian or Pacific Islander	510 (4.7%)	476 (4.8%)	34 (4.6%)	
Marital status	Married	6,219 (58.0%)	5,783 (57.9%)	436 (59.3%)	0.439
	Unmarried	4,511 (42.0%)	4,212 (42.1%)	299 (40.7%)	
Primary site	Upper third esophagus	700 (6.5%)	668 (6.7%)	32 (4.4%)	0.014
	Middle third esophagus	2,067 (19.3%)	1,911 (19.1%)	156 (21.2%)	
	Lower third esophagus	7,456 (69.5%)	6,954 (69.6%)	502 (68.3%)	
	Overlapping lesion	507 (4.7%)	462 (4.6%)	45 (6.1%)	
Histologic type	Adenocarcinoma	7,475 (69.7%)	6,904 (69.1%)	571 (77.7%)	<0.001
	Squamous-cell carcinoma	3,255 (30.3%)	3,091 (30.9%)	164 (22.3%)	
Grade	I–II	5,236 (48.8%)	4,975 (49.8%)	261 (35.5%)	<0.001
	III–IV	5,494 (51.2%)	5,020 (50.2%)	474 (64.5%)	
*N* stage	N0	4,306 (40.1%)	4,121 (41.2%)	185 (25.2%)	<0.001
	N1	4,765 (44.4%)	4,332 (43.4%)	433 (58.9%)	
	N2	1,210 (11.3%)	1,139 (11.4%)	71 (9.7%)	
	N3	449 (4.2%)	403 (4.0%)	46 (6.2%)	
Brain metastasis	No	10,562 (98.4%)	9,881 (98.9%)	681 (92.7%)	<0.001
	Yes	168 (1.6%)	114 (1.1%)	54 (7.3%)	
Liver metastasis	No	9,228 (86.0%)	8,779 (87.8%)	449 (61.1%)	<0.001
	Yes	1,502 (14.0%)	1,216 (12.2%)	286 (38.9%)	
Lung metastasis	No	9,824 (91.6%)	9,285 (92.9%)	539 (73.3%)	<0.001
	Yes	906 (8.4%)	710 (7.1%)	196 (26.7%)	

^a^ The values are given as the median, with the interquartile range in parentheses. BM, bone metastasis.

### Nomogram development for predicting BM risk

**Figure 2:**
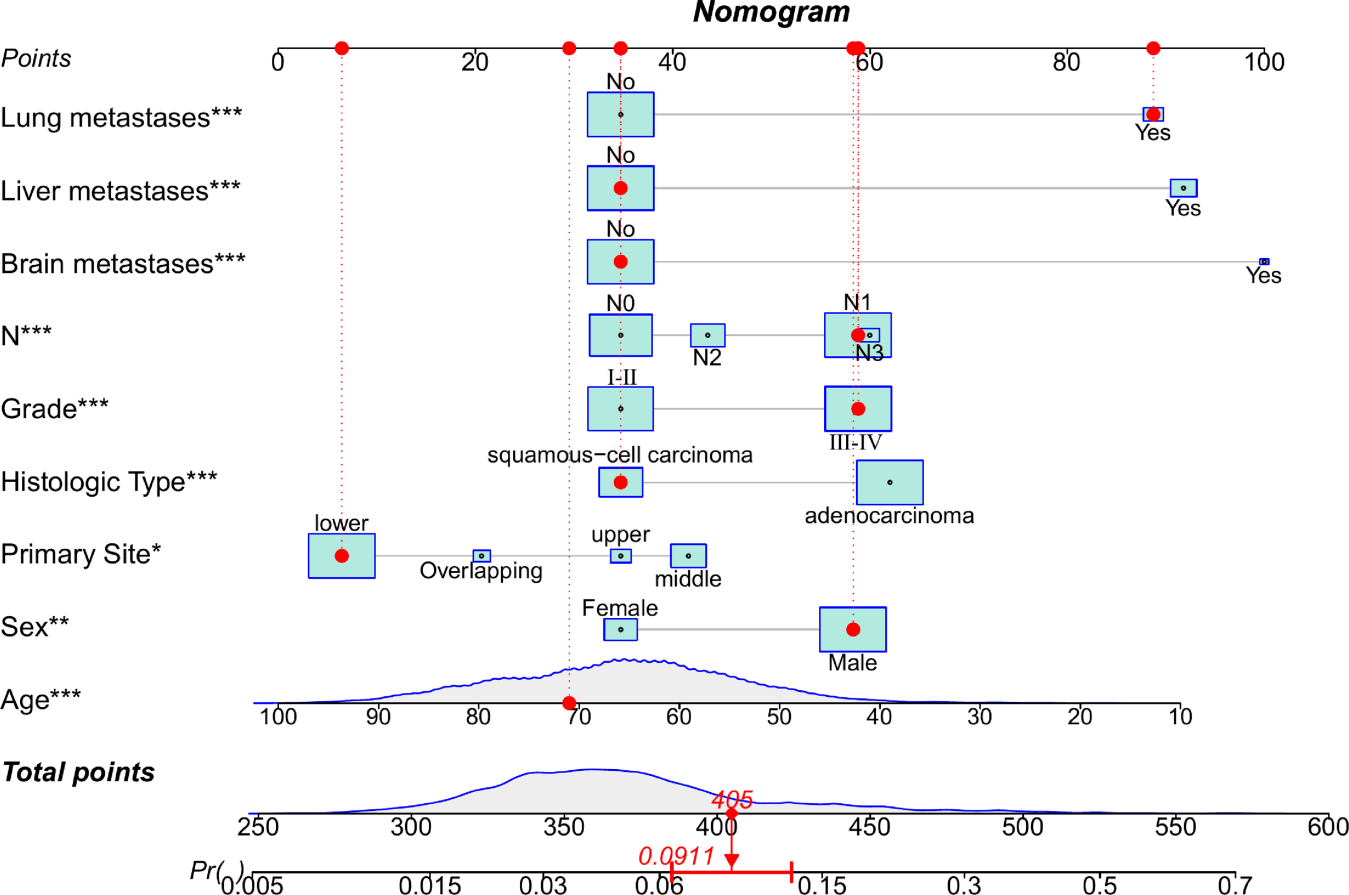
Nomogram to predict the risk of bone metastasis. A random patient was used as a demonstration. The red dot corresponds to the points of each predictor. The sum of the values for each predictor is given as total points, which corresponds to the probability of BM shown at the bottom of the chart. And the red arrow below represents the probability of bone metastasis.

The diagnostic cohort included all 10,730 patients with EC to predict the possibility of BM. Participants were randomly assigned to training (7,511 patients) and validation groups (3,219 patients). Table 2 showed the comparison of the characteristics of the two groups. No significant statistical differences were found in predictors between the two groups. The predictors were preliminarily analyzed by univariate logistic regression analyses in the training group. Statistical results displayed that the *P*-values for histologic type, primary site, age, race, sex, grade, *N* stage, and liver, lung, and brain metastases were less than 0.05, which were further identified through multivariate logistic regression analyses (Table 3). Histologic type, primary site, age, sex, tumor differentiation grade, *N* stage, and liver, lung, and brain metastases were identified as independent risk factors for BM in patients with EC. We then developed a diagnostic nomogram to predict the probability of BM based on these predictors (Figure 2). A scale can be seen at the top of the nomogram. Basic information about the patient is obtained, and by drawing a vertical line, the corresponding value of each variable on the scale can be obtained. The values of each variable are added up as a total score and the probability of occurrence of BM is obtained at the bottom of the nomogram.

**Table 2:** Comparison of characteristics between training and validation groups in patients with esophageal cancer.

Variables	Level	Training Set (*N* = 7,511)	Validation Set (*N* = 3,219)	*P* Value
Age(y)^a^		66(58,74)	66 (58,73)	0.697
Sex	Female	1,473 (19.6%)	614 (19.1%)	0.520
	Male	6,038 (80.4%)	2,605 (80.9%)	
Insurance status	Uninsured	257 (3.4%)	104 (3.2%)	0.615
	Insured	7,254 (96.6%)	3,115 (96.8%)	
Race	Black	713 (9.5%)	323 (10.0%)	0.749
	White	6,397 (85.2%)	2,723 (84.6%)	
	American Indian/Alaska Native	47 (0.6%)	17 (0.5%)	
	Asian or Pacific Islander	354 (4.7%)	156 (4.9%)	
Marital status	Married	4,355 (58.0%)	1,864 (57.9%)	0.942
	Unmarried	3,156 (42.0%)	1,355 (42.1%)	
Primary site	Upper third esophagus	495 (6.6%)	205 (6.4%)	0.851
	Middle third esophagus	1,455 (19.4%)	612 (19.0%)	
	Lower third esophagus	5,213 (69.4%)	2,243 (69.7%)	
	Overlapping lesion	348 (4.6%)	159 (4.9%)	
Histologic type	Adenocarcinoma	5,235 (69.7%)	2,240 (69.6%)	0.909
	squamous-cell carcinoma	2,276 (30.3%)	979 (30.4%)	
Grade	I–II	3,686 (49.1%)	1,550 (48.2%)	0.381
	III–IV	3,825 (50.9%)	1,669 (51.8%)	
*N* stage	N0	2,978 (39.6%)	1,328 (41.3%)	0.105
	N1	3,351 (44.6%)	1,414 (43.9%)	
	N2	877 (11.7%)	333 (10.3%)	
	N3	305 (4.1%)	144 (4.5%)	
Brain metastasis	No	7,394 (98.4%)	3,168 (98.4%)	0.919
	Yes	117 (1.6%)	51 (1.6%)	
Liver metastasis	No	6,485 (86.3%)	2,743 (85.2%)	0.123
	Yes	1,026 (13.7%)	476 (14.8%)	
Lung metastasis	No	6,884 (91.7%)	2,940 (91.3%)	0.585
	Yes	627 (8.3%)	279 (8.7%)	

^a^ The values are given as the median, with the interquartile range in parentheses (The distribution of age is not normal in the whole cohort).

**Table 3:** Univariate and multivariate logistic regression analyses in esophageal cancer patients with bone metastasis in the training group.

Variables	Univariate		Multivariate	
	OR (95% CI)	*P* Value	OR (95% CI)	*P* Value
Age	0.975 (0.967–0.982)	<0.001	0.981 (0.973–0.990)	<0.001
Sex				
Female	Reference			
Male	1.833 (1.394–2.410)	<0.001	1.569 (1.172–2.099)	0.002
Insurance status				
Uninsured	Reference			
Insured	0.656 (0.430–1.001)	0.051		
Race				
White	Reference			
Black	0.888 (0.643–1.228)	0.473	0.980 (0.676–1.420)	0.914
American Indian/Alaska Native	2.839 (1.319–6.114)	0.008	2.137 (0.929–4.916)	0.074
Asian or Pacific Islander	0.917 (0.589–1.428)	0.702	0.959 (0.595–1.543)	0.862
Marital status				
Married	Reference			
Unmarried	0.942 (0.784–1.133)	0.527		
Primary site				
Upper third esophagus	Reference			
Middle third esophagus	1.598 (1.024–2.495)	0.039	1.137 (0.713–1.813)	0.589
Lower third esophagus	1.295 (0.853–1.966)	0.224	0.581 (0.361–0.935)	0.025
Overlapping lesion	1.709 (0.983–2.973)	0.058	0.762 (0.419–1.385)	0.372
Histologic type				
Squamous–cell carcinoma	Reference			
Adenocarcinoma	1.583 (1.275–1.966)	<0.001	1.668 (1.244–2.235)	0.001
Grade				
I–II	Reference			
III–IV	1.871 (1.548–2.26)	<0.001	1.584 (1.298–1.934)	<0.001
*N* stage				
N0	Reference			
N1	2.218 (1.79–2.748)	<0.001	1.583 (1.263–1.985)	<0.001
N2	1.311 (0.932–1.844)	0.120	1.192 (0.838–1.694)	0.329
N3	2.561 (1.696–3.867)	<0.001	1.631 (1.055–2.521)	0.028
Brain metastasis				
No	Reference			
Yes	6.86 (4.595–10.242)	<0.001	3.452 (2.217–5.377)	<0.001
Liver metastasis				
No	Reference			
Yes	4.736 (3.904–5.745)	<0.001	2.985 (2.401–3.712)	<0.001
Lung metastasis				
No	Reference			
Yes	4.904 (3.948–6.093)	<0.001	2.803 (2.194–3.582)	<0.001

### Performance and validation of the diagnostic nomogram

**Figure 3:**
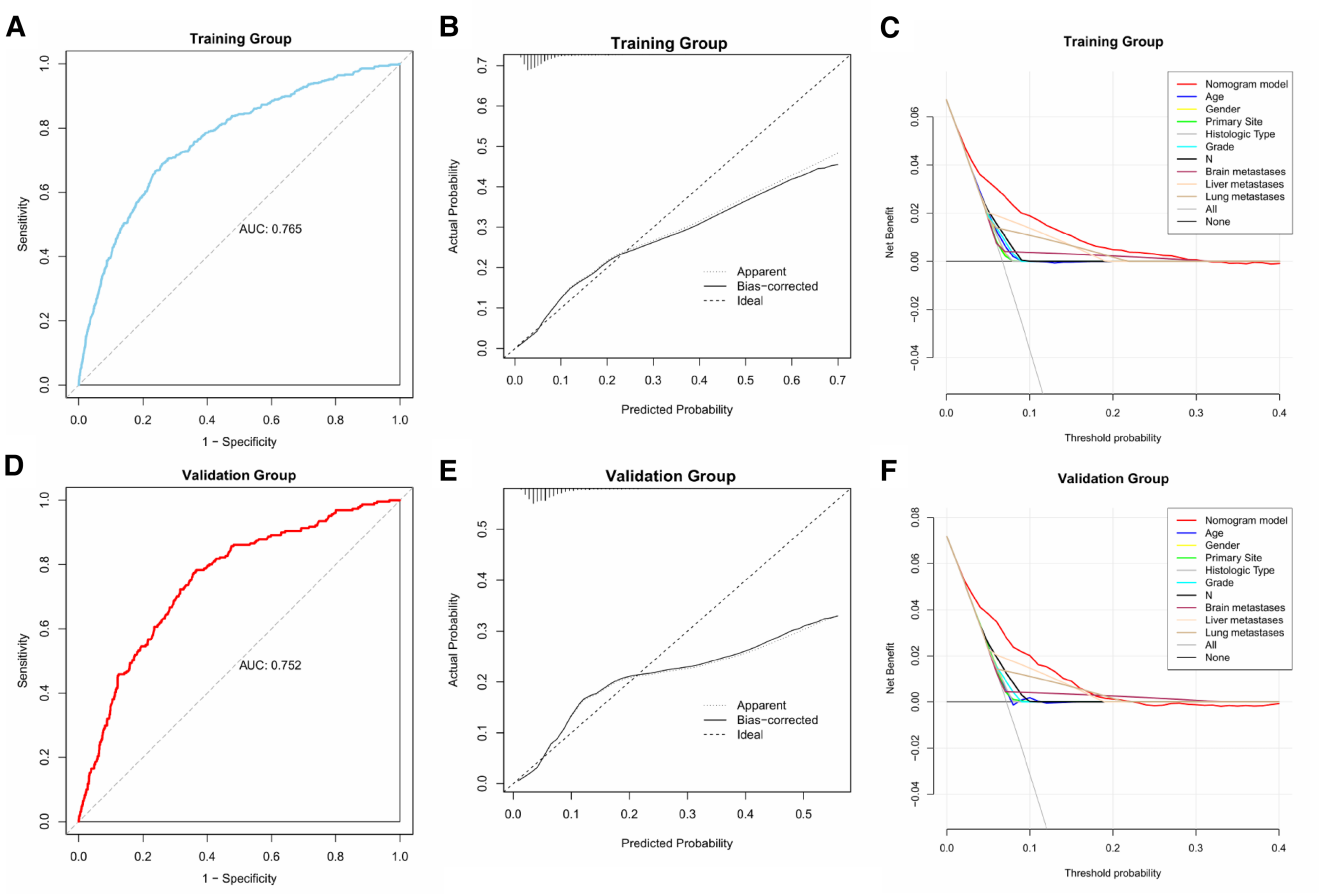
ROC, calibration, and DCA curves of the predictive model in the training and validation groups.

**Figure 4:**
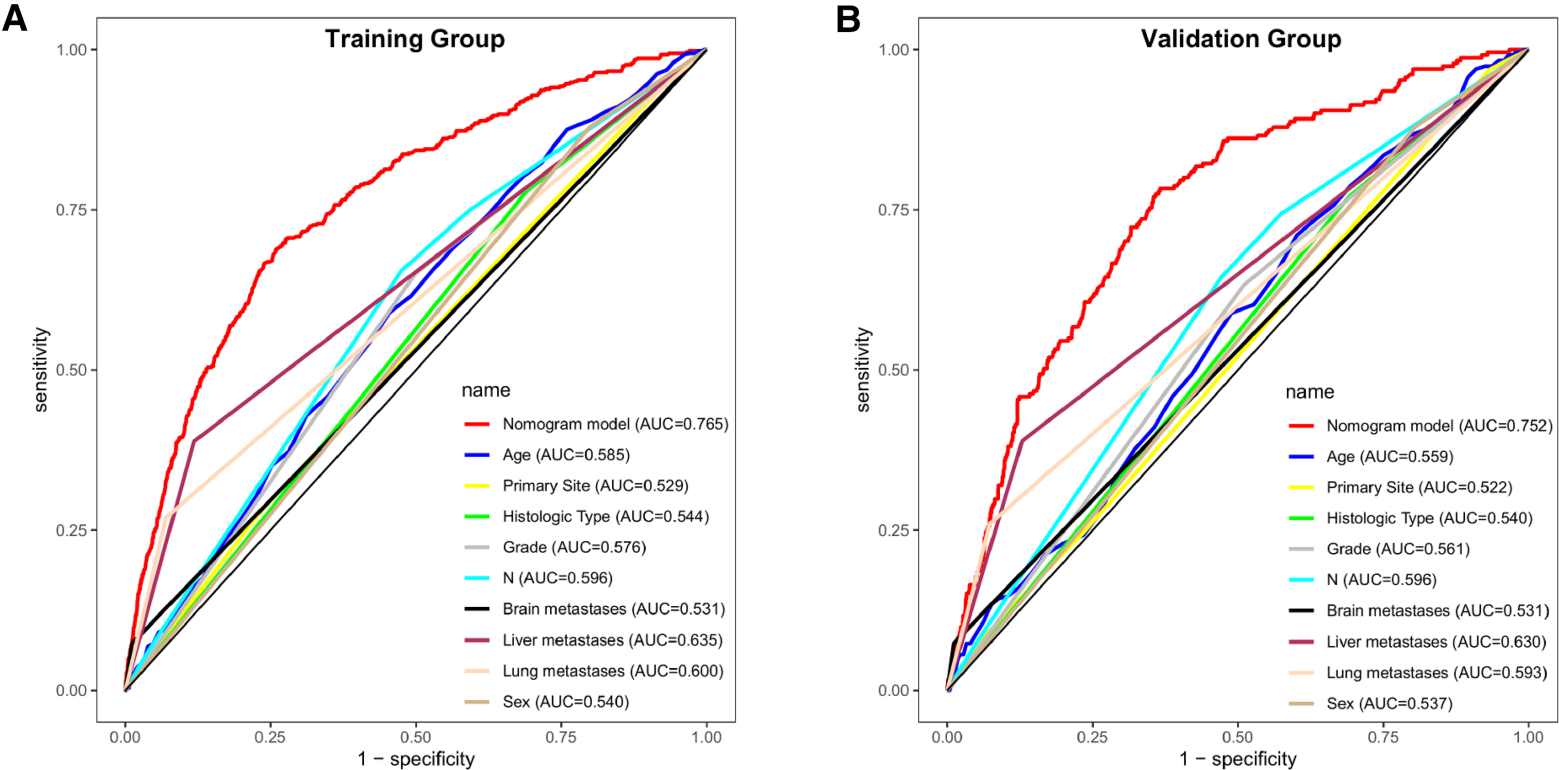
ROC curves and AUC values of predictive nomograms and each predictor in the training group (**A**) and validation group (**B**).

We plotted the ROC, calibration, and DCA curves to assess the performance of the diagnostic nomogram in predicting BM in the training group (Figure 3). The AUC value was 0.765 (95% CI: 0.743–0.783), and the AUC of this model was larger than that of any single predictor (Figure 4), representing better disease-prediction ability. The calibration curve displayed good consistency between the predicted and actual probabilities. DCA curves suggested that the nomogram possessed good clinical net benefits as an accurate tool for BM assessment and that the model was more valuable than any single predictor.

The same assessment method was used for the validation group. The AUC value of the diagnostic nomogram was 0.752 (95% CI: 0.721–0.784), which was greater than that of any single predictor as well (Figure 4). The calibration curve was consistent between the actual and predicted results, meanwhile, the predictive nomogram also possessed good clinical application value in the DCA curve, outperforming all individual predictors (Figure 3).

### Nomogram development for prognosis in patients with EC and BM

In the survival cohort, 659 patients with EC and BM were included in total (11 patients were excluded due to incomplete treatment records, and 65 patients were excluded due to less than 1 month survival). In a 3:7 ratio, 198 and 461 participants were divided into the validation and training groups, respectively. Both patients in the training and validation groups possessed a median OS of only 5 months. No significant statistical differences were found in clinicopathological and treatment data between the validation and training groups (Table 4).

**Table 4:** Comparison of characteristics between training and validation groups in esophageal cancer patients with bone metastasis.

Variables	Level	Training Set (*N* = 461)	Validation Set (*N* = 198)	*P* Value
Age(y)^a^		63.1 ± 10.3	61.5 ± 10.8	0.073
Sex	Female	51 (11.1%)	29 (14.6%)	0.197
	Male	410 (88.9%)	169 (85.4%)	
Insurance status	Uninsured	22 (4.8%)	5 (2.5%)	0.182
	Insured	439 (95.2%)	193 (97.5%)	
Race	Black	43 (9.3%)	18 (9.1%)	0.381
	White	386 (83.7%)	172 (86.9%)	
	American Indian/Alaska Native	6 (1.3%)	3 (1.5%)	
	Asian or Pacific Islander	26 (5.7%)	5 (2.5%)	
Marital status	Married	280 (60.7%)	114 (57.6%)	0.448
	Unmarried	181 (39.3%)	84 (42.4%)	
Primary site	Upper third esophagus	20 (4.3%)	8 (4.1%)	0.158
	Middle third esophagus	97 (21.1%)	43 (21.7%)	
	Lower third esophagus	312 (67.7%)	142 (71.7%)	
	Overlapping lesion	32 (6.9%)	5 (2.5%)	
Histologic type	squamous–cell carcinoma	109 (23.6%)	39 (19.7%)	0.266
	adenocarcinoma	352 (76.4%)	159 (80.3%)	
Grade	I–II	179 (38.8%)	65 (32.8%)	0.144
	III–IV	282 (61.2%)	133 (67.2%)	
*N* stage	N0	116 (25.2%)	45 (22.7%)	0.735
	N1	270 (58.6%)	121 (61.1%)	
	N2	49 (10.6%)	18 (9.1%)	
	N3	26 (5.6%)	14 (7.1%)	
Brain metastasis	No	430 (93.3%)	181 (91.4%)	0.399
	Yes	31 (6.7%)	17 (8.6%)	
Liver metastasis	No	282 (61.2%)	122 (61.6%)	0.914
	Yes	179 (38.8%)	76 (38.4%)	
Lung metastasis	No	342 (74.2%)	144 (72.7%)	0.696
	Yes	119 (25.8%)	54 (27.3%)	
Surgery	No	454 (98.5%)	195 (98.5%)	0.997
	Yes	7 (1.5%)	3 (1.5%)	
Radiotherapy	No	191 (41.4%)	77 (38.9%)	0.542
	Yes	270 (58.6%)	121 (61.1%)	
Chemotherapy	No	138 (29.9%)	67 (33.8%)	0.321
	Yes	323 (70.1%)	131 (66.2%)	

^a^ The values are given as the mean and the standard deviation.

**Figure 5:**
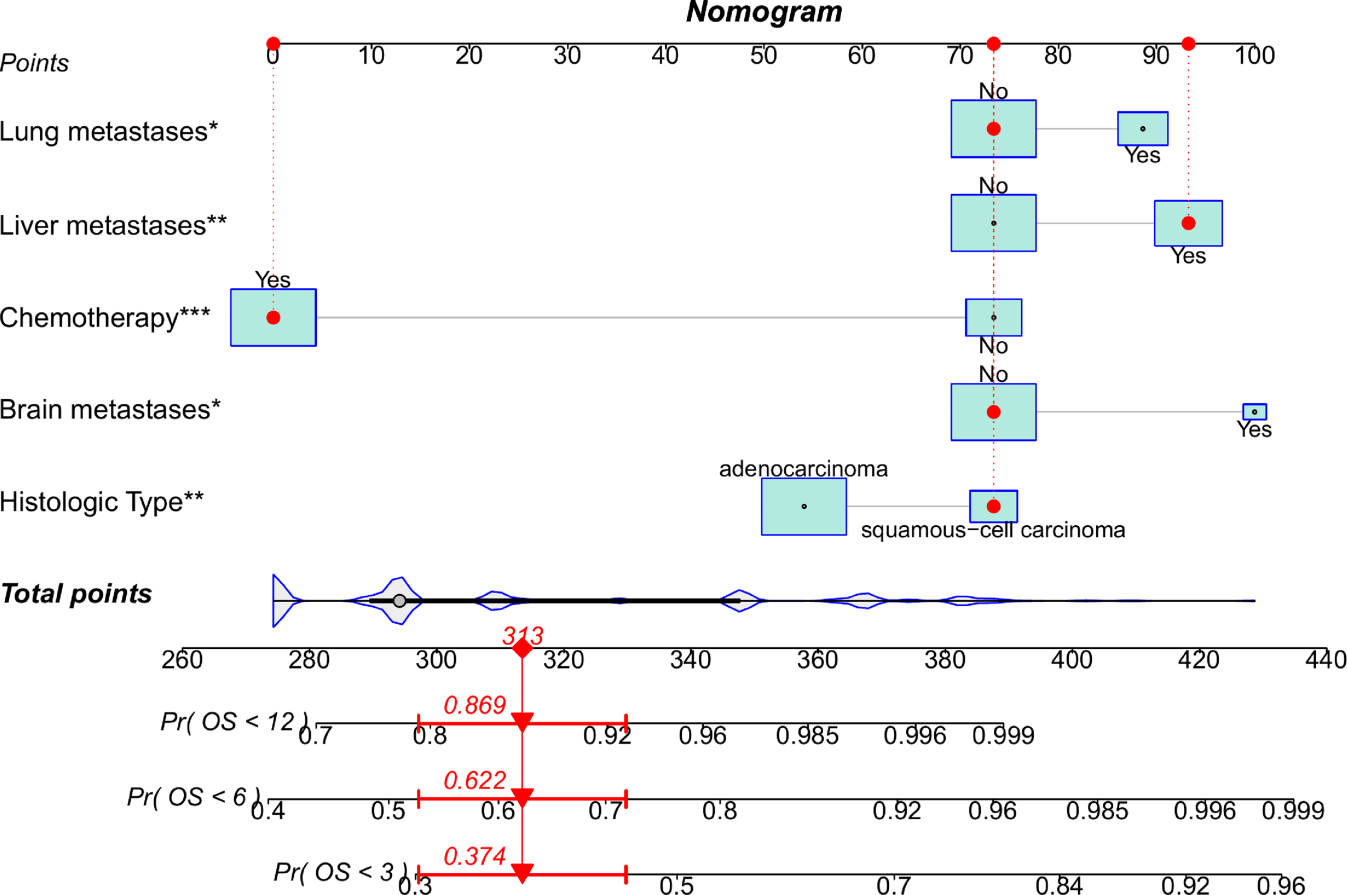
Nomogram to predict survival probability of EC patients with BM. A random patient was used as a demonstration. The red dot corresponds to the points of each predictor, and the red arrow below represents the probability of overall survival at 3, 6, and 12 months.

In the training group, 15 variables were analyzed through univariate Cox regression analysis (Table 5). Age, marital status, histologic type, brain, liver, and lung metastases, surgery, and chemotherapy possessed the *P* values less than 0.05, which were analyzed ulteriorly using multivariate Cox regression analysis. The results demonstrated that SCC, without chemotherapy, with brain, lung, and liver metastases had statistically significant effects on the OS of patients with EC and BM. According to the above outcome, we plotted a prognostic nomogram for predicting survival probability at 3, 6, and 12 months for those patients (Figure 5).

**Table 5:** Univariate and multivariate cox regression analyses in esophageal cancer patients with bone metastasis.

Variables	Univariate		Multivariate	
	HR (95% CI)	*P* Value	HR (95% CI)	*P* Value
Age	1.010 (1.001–1.020)	0.036	1.002 (0.993–1.012)	0.604
Sex				
Female	Reference			
Male	1.205 (0.890–1.632)	0.227		
Insurance status				
Uninsured	Reference			
Insured	0.652 (0.424–1.002)	0.051		
Race				
White	Reference			
Black	1.338 (0.975–1.838)	0.072		
American Indian/Alaska Native	1.331 (0.593–2.986)	0.488		
Asian or Pacific Islander	0.909 (0.602–1.375)	0.653		
Marital status				
Married	Reference			
Unmarried	1.495 (1.234–1.811)	<0.001	1.170 (0.957–1.430)	0.126
Primary site				
Upper third esophagus	Reference			
Middle third esophagus	1.066 (0.651–1.747)	0.799		
Lower third esophagus	0.924 (0.581–1.471)	0.740		
Overlapping lesion	1.335 (0.751–2.374)	0.325		
Histologic type				
Squamous-cell carcinoma	Reference			
Adenocarcinoma	0.732 (0.587–0.913)	0.006	0.745 (0.586–0.948)	0.016
Grade				
I–II	Reference			
III–IV	1.156 (0.955–1.399)	0.138		
*N* stage				
N0	Reference			
N1	0.959 (0.769–1.195)	0.706		
N2	0.809 (0.576–1.137)	0.222		
N3	0.853 (0.545–1.336)	0.488		
Brain metastasis				
No	Reference			
Yes	1.640 (1.136–2.369)	0.008	1.525 (1.050–2.214)	0.027
Liver metastasis				
No	Reference			
Yes	1.212 (1.001–1.468)	0.049	1.362 (1.118–1.661)	0.002
Lung metastasis				
No	Reference			
Yes	1.395 (1.127–1.727)	0.002	1.260 (1.003–1.582)	0.047
Surgery				
No	Reference			
Yes	0.406 (0.168–0.984)	0.046	0.470 (0.192–1.150)	0.098
Chemotherapy				
No	Reference			
Yes	0.321 (0.260–0.395)	<0.001	0.322 (0.258–0.401)	<0.001
Radiotherapy				
No	Reference			
Yes	0.853 (0.706–1.030)	0.099		

### Performance and validation of the prognostic nomogram

**Figure 6:**
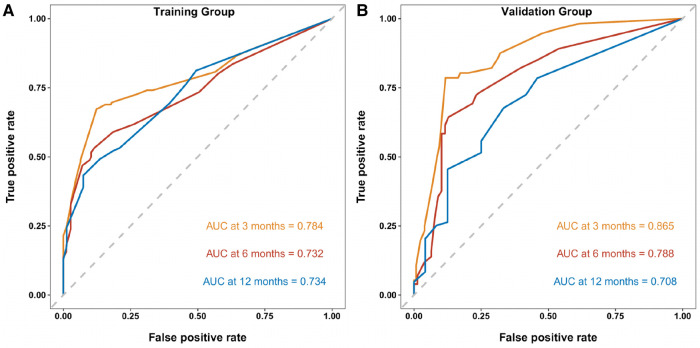
ROC curves of the prognostic model at 3, 6, and 12 months in the training group (**A**), and ROC curves of the prognostic model at 3, 6, and 12 months in the validation group (**B**).

**Figure 7:**
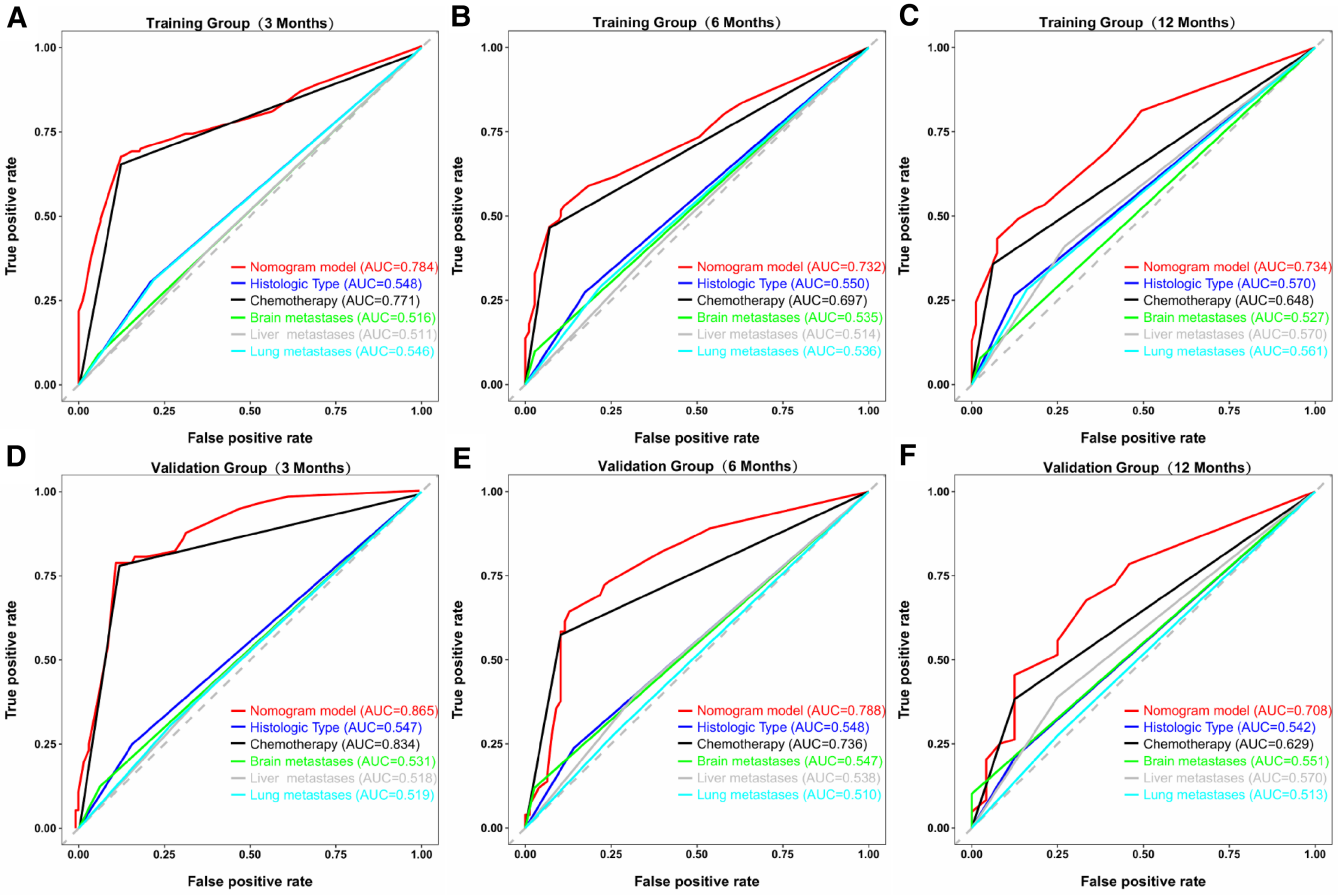
ROC curves of the prognostic model and each predictor at 3 (**A**), 6 (**B**), and 12 months (**C**) in the training group, and at 3 (**D**), 6 (**E**), and 12 months (**F**) in the validation group. The AUC values of the nomogram were larger than all single predictors at 3, 6, and 12 months in both groups.

**Figure 8:**
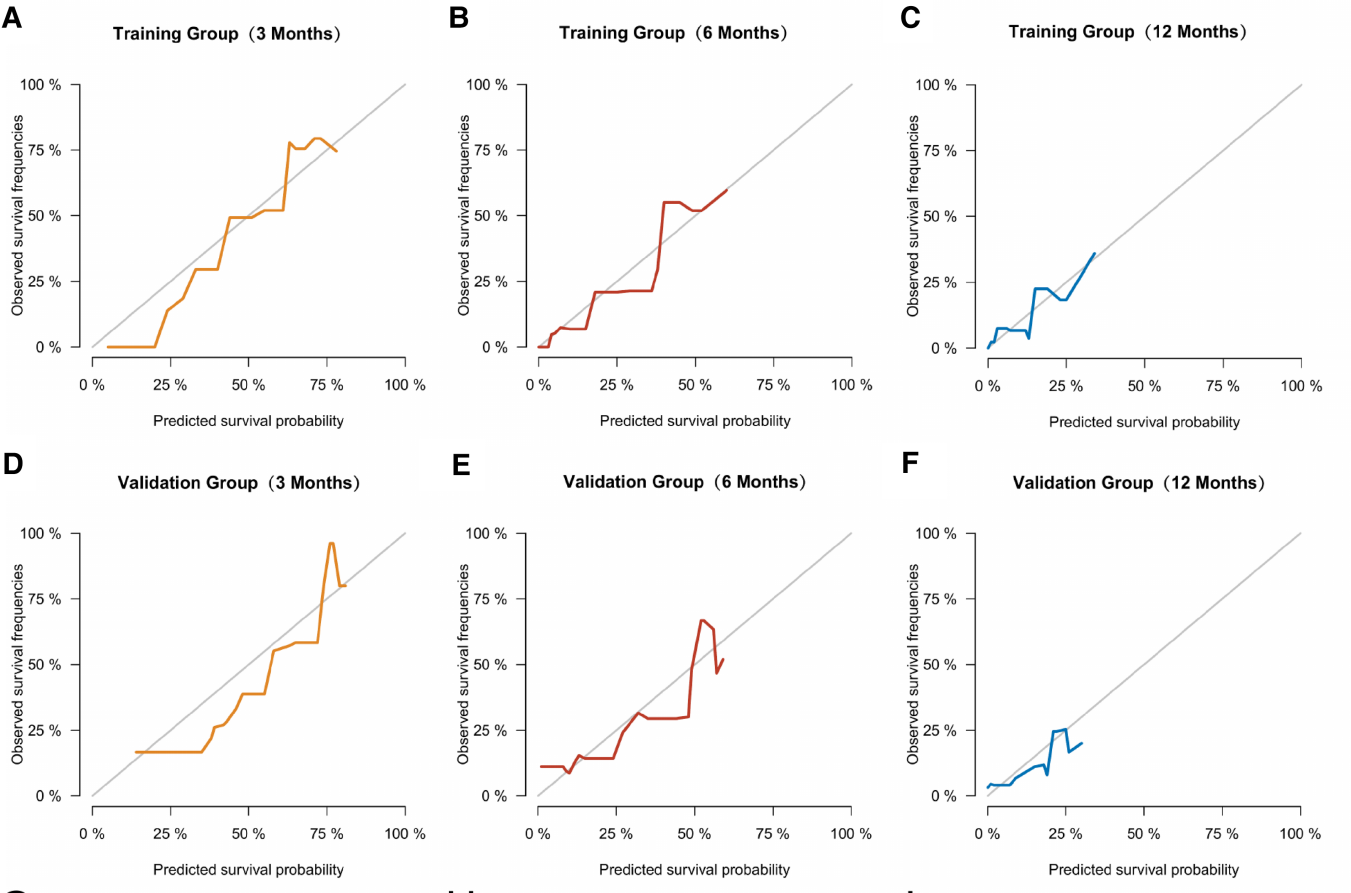
Calibration curves of the prognostic model at 3 (**A**), 6 (**B**), and 12 months (**C**) in the training group, and at 3 (**D**), 6 (**E**), and 12 months (**F**) in the validation group.

**Figure 9:**
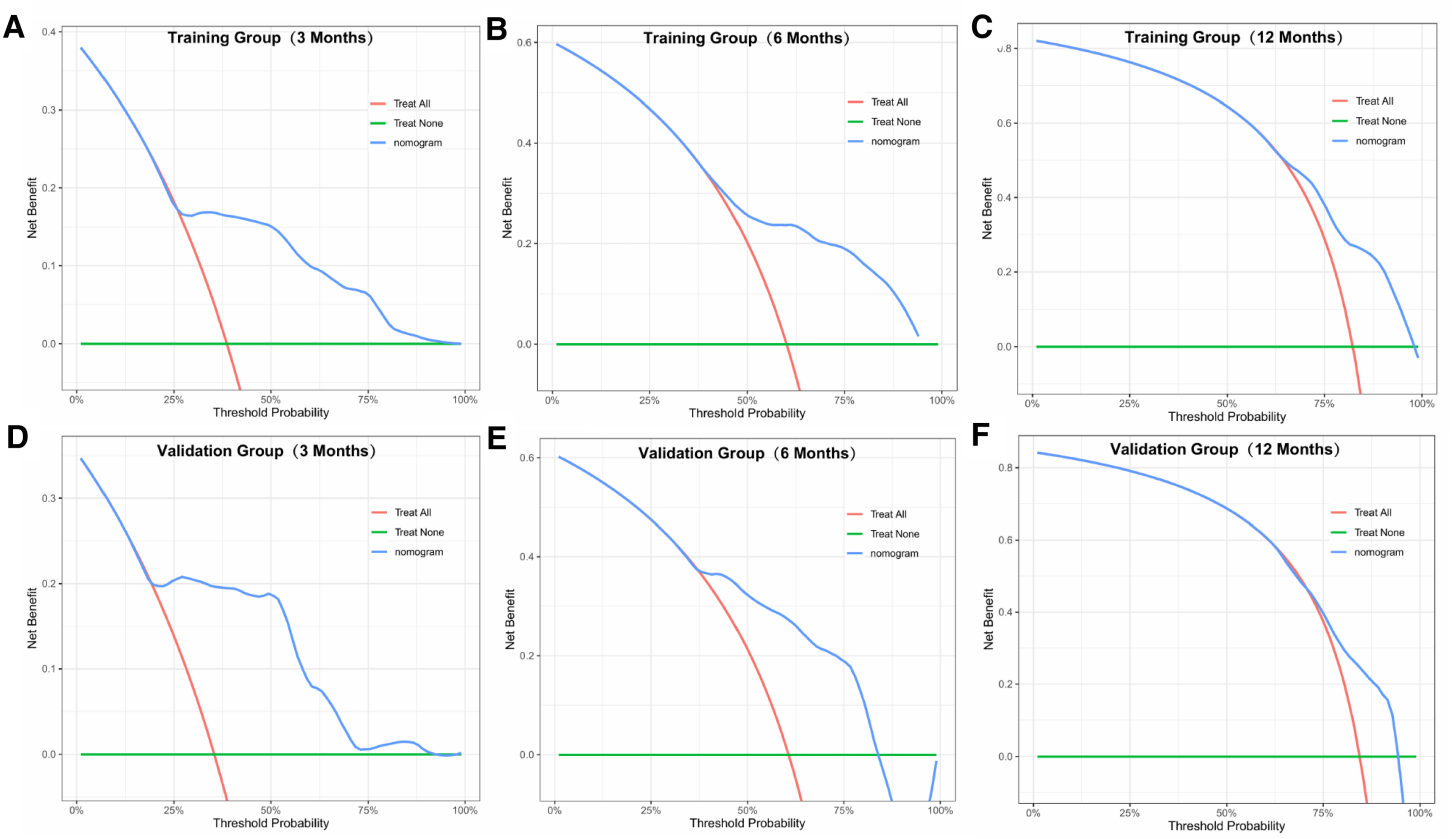
DCA curves of the prognostic model at 3 (**A**), 6 (**B**), and 12 months (**C**) in the training group, and at 3 (**D**), 6 (**E**), and 12 months (**F**) in the validation group.

**Figure 10:**
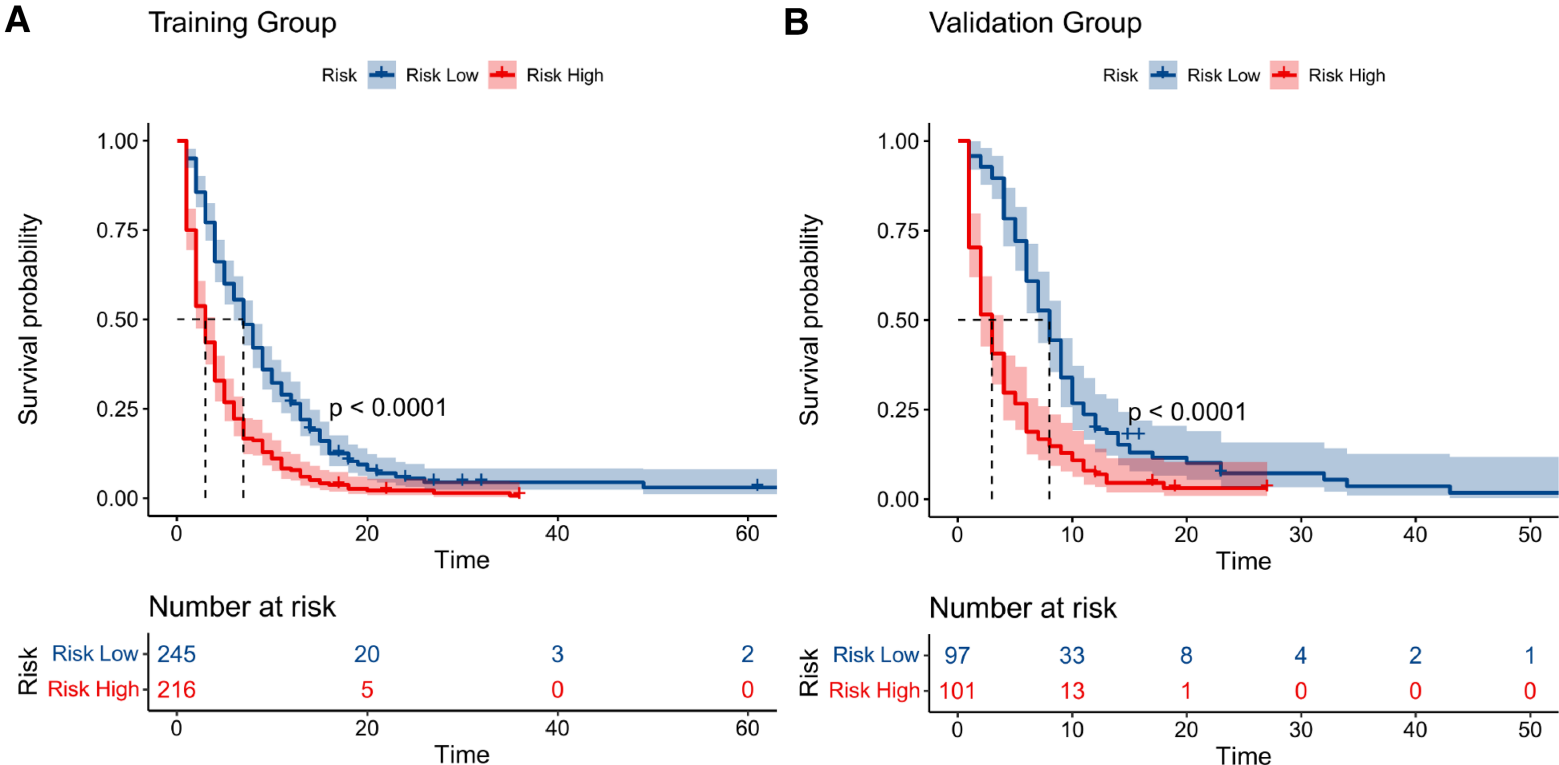
Kaplan–meier curves of high- and low-risk patients in the training group (**A**) and validation group (**B**). Patients in the low-risk group had a higher survival probability than those in the high-risk group (*P* < 0.0001) in both cohorts.

Similarly, we used ROC, calibration, and DCA curves to evaluate the predictive efficiency of the prognostic nomogram. The AUC values of the nomogram at 3, 6, and 12 months in the training group were 0.784 (95% CI, 0.732–0.837), 0.732 (95% CI, 0.687–0.778), and 0.734 (95% CI, 0.682–0.786), respectively. The AUC values in the validation group at 3, 6, and 12 months were 0.865 (95% CI, 0.810–0.920), 0.788 (95% CI, 0.719–0.857), and 0.708 (95% CI, 0.602–0.814), respectively (Figure 6). Compared with the individual prognostic factors, the prognostic nomogram had larger AUC values at 3, 6, and 12 months in both the training and validation group (Figure 7). Calibration curves of the prognostic model at 3, 6, and 12 months in both the training and validation group were fitted to the 45° line, suggesting high consistency between the predicted survival probability and observed living status in both groups (Figure 8). DCA curves of the nomogram at 3, 6, and 12 months are shown in Figure 9. The prognostic model had a high net benefit in patients with BM at 3 months, followed by 6 and 12 months. The prognostic nomogram showed a significant positive net benefit over a wide range of death risks in either the training or validation group, suggesting that the prediction model has good clinical application value in patients with BM. Moreover, patients with EC and BM were assigned to high-risk (score > 294.0 points) and low-risk cohorts (score ≤ 294.0 points) based on the total points calculated by the prognostic nomogram. The K-M curve indicated a significantly higher survival rate for low-risk group patients (Figure 10).

## Discussion

BM is one of the most common metastatic pathways in advanced EC, and usually predicts poorer prognosis and shorter survival expectancy (15). The incidence of BM in patients with EC was 6.8% in our study, which was consistent with the range of 5.2%–7.7% reported in previous studies (3, 12). However, the incidence of BM is often underestimated because imaging methods to detect BM, including bone scintigraphy and positron emission tomography-computed tomography, are relatively expensive and not routinely performed (15). Moreover, the symptoms of BM are not significant in the early stage and are easily masked by other symptoms, which also leads to underestimation (14). Therefore, the predictive nomogram used in this study is conducive for predicting the probability of BM and timely intervention in high-risk patients according to the probability of metastasis to prevent disease progression. Traditional TNM staging can only roughly predict prognosis, whereas the survival time of patients can be quantitatively predicted using the prognostic nomogram. Therefore, the nomogram has high clinical decision-making value for patients with both suspected and confirmed BM, and the treatment strategy can be adjusted through survival prediction, thereby improving prognosis. To the best of our knowledge, this is the first time a model that can quantitatively predict the risk or prognosis of BM for EC has been established.

In this study, histologic type, primary site, age, sex, tumor differentiation grade, N stage, and lung, liver, and brain metastases were found to be independent risk factors for BM in patients with EC. Unexpectedly, older patients with EC tended to have a lower probability of BM in this cohort. This conclusion is in accordance with the finding by Qin et al. which determined that patients aged 51 to 60 years had a higher risk of BM compared with patients aged 71 to 80 years (15). This phenomenon also occurs in liver metastasis of EC (10), and capillary sclerosis may be an important factor in reducing distant organ metastasis in older adults (7). In addition, we found men were more likely to develop BM from EC than women. This phenomenon may be related to higher rates of smoking and drinking among men, which are the risk factors for BM (27, 28). Among all primary sites of EC, middle esophageal carcinoma had the highest rate of BM. This finding may be because the middle esophagus is anatomically closest to spine and shares the most blood supply with the spine (8, 29, 30). Because the blood flow of the spinal vein runs slowly and is interconnected, once the pressure of the thoracic or abdominal cavity increases, the tumor embolus can directly enter the vertebral vein system and cause metastasis (29). Among all the risk factors, brain metastasis had the most significant impact on BM development, followed by liver and lung metastases. The study by Zhang et al. had the same results (14), probably owing to the fact that cancer has already widely spread through the blood and caused damage when these metastases occur (31, 32). Moreover, AC contributed more to the development of BM than SCC. Another study including 9,934 stage I–IV patients with EC suggested that the AC subtype was more likely to cause BM than SCC tumors (7). The exact mechanism for this metastasis remains unclear, several researchers have pointed out that high expression of sorting nexin 3, sphingosine-1-phosphate receptor 2, and toll-like receptor 9 may explain the high invasiveness of AC in patients with EC (33–35).

We established a predictive nomogram to evaluate the probability of BM in patients with EC. AUC values of this model in the training and validation groups were 0.765 and 0.752, respectively, indicating that the nomogram had good prediction ability. Notably, the predictive model possessed great clinical application potential according to the calibration and DCA curves in either the training or validation groups.

In the prognostic prediction nomogram, histological type, chemotherapy, and brain, lung, and liver metastases were found to be independent prognostic risk factors for patients with BM. Compared with surgery and radiotherapy, chemotherapy plays a more important role in prognosis. Qiu et al. found that older patients who underwent chemotherapy had better survival than those who did not, regardless of whether the patient received surgery or radiation (11). The National Comprehensive Cancer Network (NCCN) guidelines recommend chemotherapy as first-line treatment for patients with metastaticEC, with targeted therapy combined with chemotherapy as a second-line treatment option (36). In addition, it has been shown that zoledronate decreases the incidence of skeletal-related events in patients with BMs, and thus may be associated with improved survival (37). However, due to the limitations of the SEER database, we were unable to validate the effectiveness of these therapeutic measures in patients with EC and BM.

Regarding histological type, we found that patients with EC and SCC tend to have worse OS than patients with AC. Zhang et al. also found that patients with esophageal AC had a higher risk of BM, whereas patients with esophageal SCC had a worse prognosis after BM (14). To date, the mechanisms related to the prognosis of SCC remain unknown; however, at the molecular level, abnormal expression of FAM3C, AKAP8L, and E2F5 may be associated with poor prognosis (38–40).

Patients with EC and BM had poor prognosis and relatively short median OS of only 5 months. Therefore, we established a prognostic nomogram to quantitatively predict the survival at 3, 6, and 12 months in patients with EC and BM. The AUC values and calibration curves indicated that the prognostic model had good predictive ability in patients with EC and BM. A similar situation can be seen in the DCA curve, where the model had a high net benefit for patients with BM. Furthermore, the high-risk patients screened by the prognostic nomogram had dramatically worse survival rates in the K-M curves, reflecting the good discriminatory ability of the model.

This study had a few limitations. First, since the information was extracted from the SEER database, the predictors were limited to the demographic and disease indicators recorded in the database, which may make it difficult to improve the accuracy of the prediction models. Moreover, as patients with EC and BM had a median OS of only 5 months in this study, few patients survived for more than a full year, so the predictive ability for the long-term survival of this model is relatively limited. Finally, internal validation was performed in this study and showed good predictive power of the nomograms, however, given the ethnic differences and the high incidence of EC in East Asia, we will collect information from a large number of patients with EC in China to perform external validation to better validate and explain the findings of this study in the future. Despite these limitations, this study has developed models that can quantitatively and individually predict the risk and prognosis of BM in patients with EC, which can greatly improve disease surveillance and clinical decision making.

## Conclusions

This study determined that histologic type, primary site, age, sex, tumor differentiation grade, N stage, and liver, lung, and brain metastases were independent risk factors for BM in patients with EC. Moreover, in patients with EC and BM, histological type, chemotherapy, and brain, lung, and liver metastases were identified as prognostic risk factors. We established and validated two novel nomograms in patients with EC to quantitatively predict the risk and prognosis of BM. These prediction models can effectively help patients and clinicians in disease surveillance and clinical decision-making based on their good accuracy and reliability.

## Data Availability

Publicly available datasets were analyzed in this study. This data can be found here: https://seer.cancer.gov/.
